# Subcostal approach using the single-port robotic system for a giant ganglioneuroma in a child

**DOI:** 10.1016/j.xjtc.2025.04.010

**Published:** 2025-04-22

**Authors:** Shinji Kaneda, Koji Kawaguchi, Atsushi Ito, Daisuke Ito, Teruhisa Kawaguchi, Akira Shimamoto, Motoshi Takao

**Affiliations:** a Department of Thoracic Surgery, Mie University Hospital, Tsu, Mie Prefecture, Japan; b Department of Clinical Research Centre, Mie University Hospital, Tsu, Mie Prefecture, Japan; c Department of Thoracic Surgery, Matsusaka City Hospital, Matsusaka, Mie Prefecture, Japan

**Figure:**
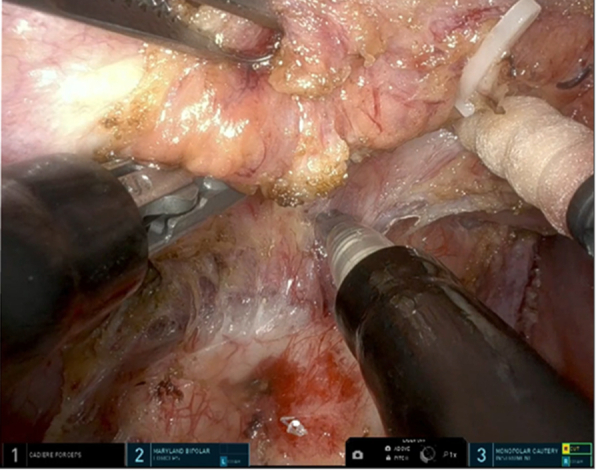
A surgical scene with the da Vinci SP (Intuitive).

**Central Message**

Surgery using the subcostal approach with the da Vinci SP (Intuitive) for giant nerve tumors in pediatric patients. It was effective and safe for thoracic surgery in children.

## Clinical Summary

**Figure 1:**
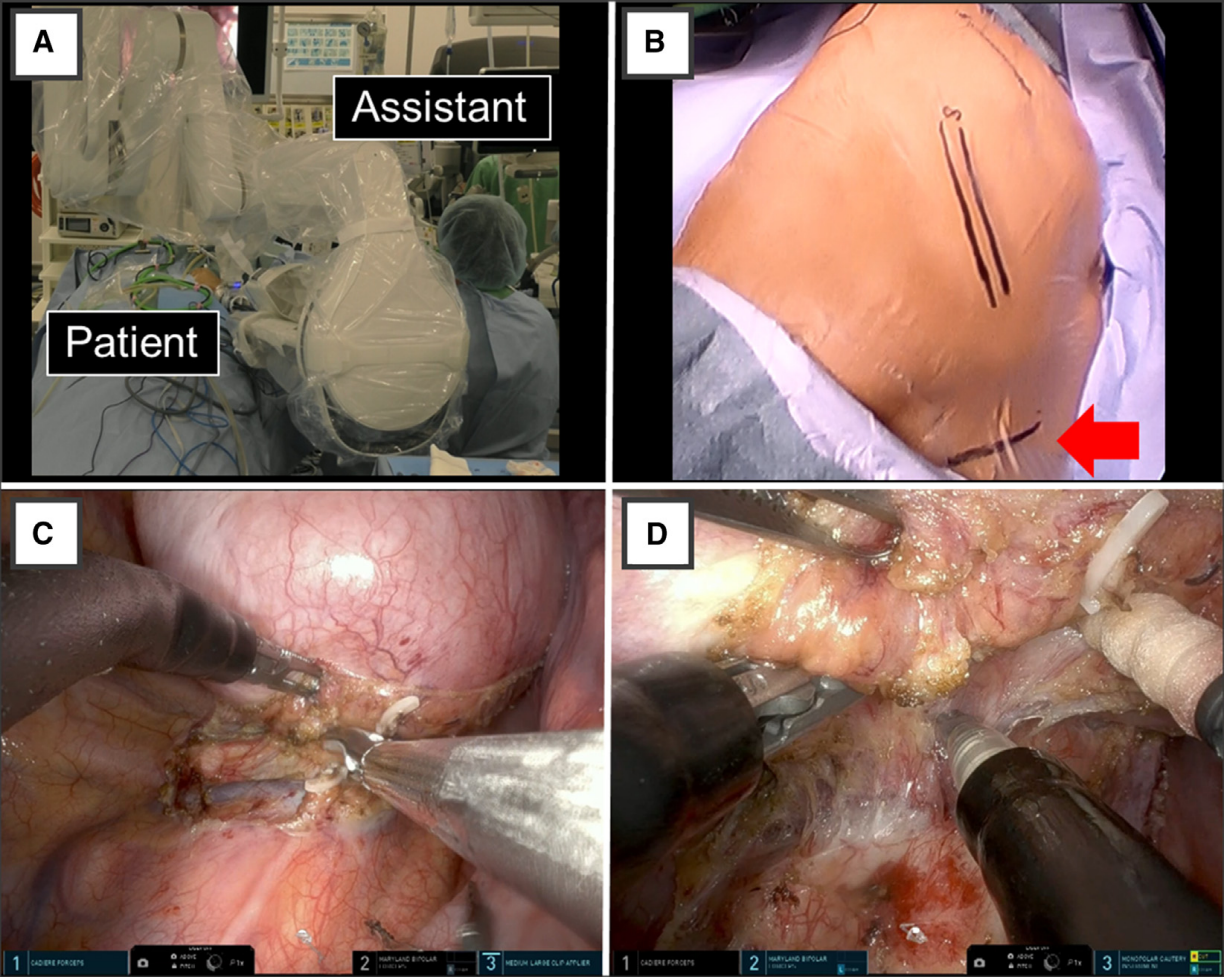
A, The surgical scene. B, Skin incision (*arrow*). C, The use of LigaSure (Medtronic). D, The tumor was lifted and the base was debrided.

**Figure 2:**
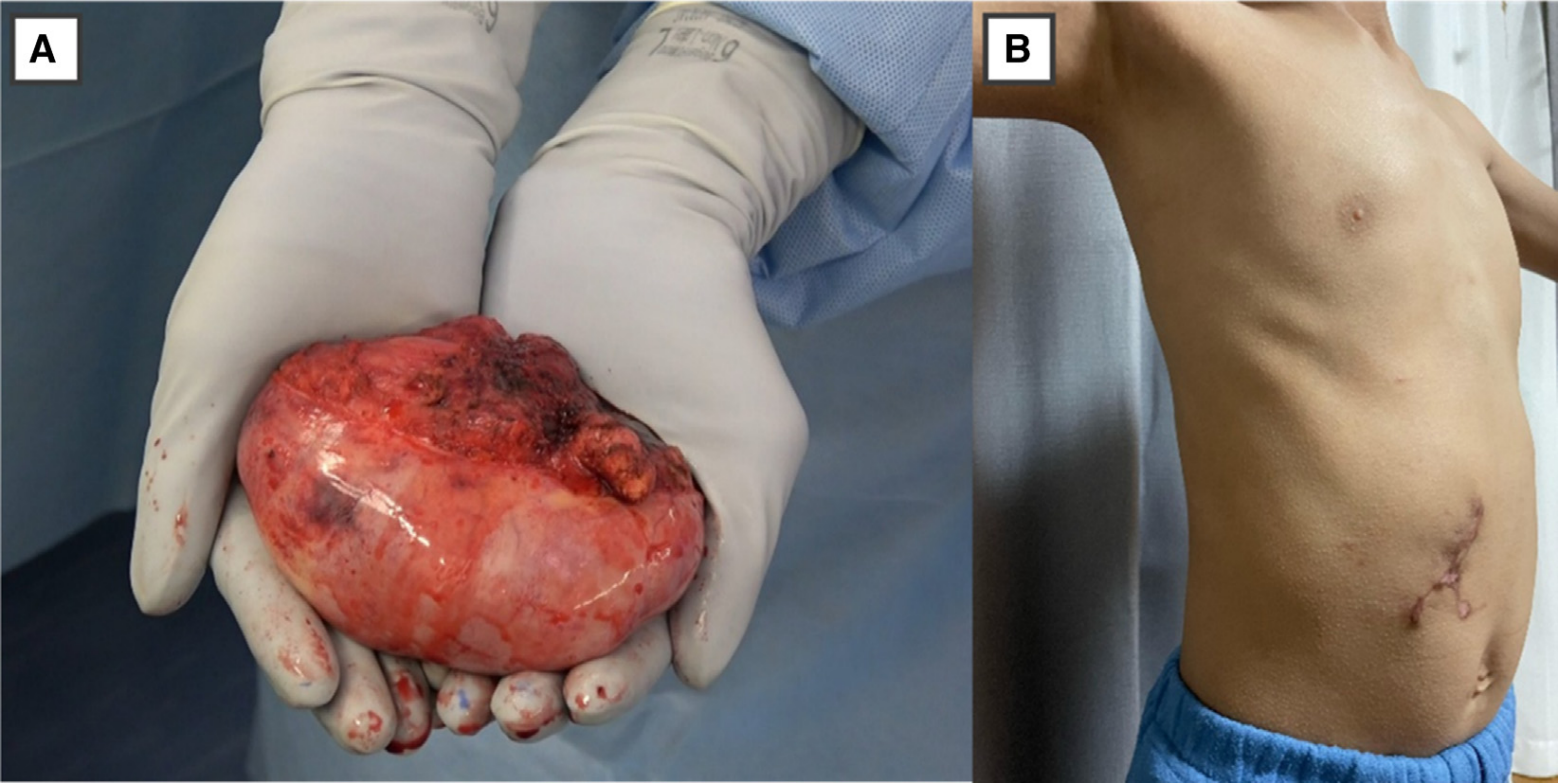
A, Macroscopic findings of the tumor. B, The patient's incision on postoperative day 23.

The patient was an 8-year-old boy (height, 125 cm; body weight, 22.5 kg) who presented with fever. Computed tomography revealed a 10.3 × 7.8 cm mediastinal tumor. The preoperative diagnosis, based on magnetic resonance imaging findings, was suspected gangliocytoma. Tumor resection was planned using the da Vinci SP (Intuitive) (Video 1 and Figure 1, *A*). The surgery was performed with isolated lung ventilation and carbon dioxide insufflation at 8 mm Hg in the left lateral position. A 4.0-cm skin incision was made in the subcostal area (Figure 1, *B*) and an S-size access port was used. The tumor was covered by pleura and did not adhere to the lungs. The sympathetic trunk was involved in the tumor and was hemoclipped and separated using a vessel-sealing system (Figure 1, *C*). The tumor was located in the sixth intercostal space. The intercostal nerve was thickened and contiguous with the tumor, and a gangliocytoma arising from the intercostal nerve was suspected. The intercostal nerve was dissected to what appeared to be normal, and a frozen biopsy confirmed negative margins. The skin incision was extended to 5.5 cm and the tumor was removed. A 20Fr thoracic drain was inserted through the subcostal incision and the wound was closed. The operative time was 250 minutes, the console time was 187 minutes, and blood loss was minimal. A subcostal transversus abdominis plane block was performed before the patient was awakened from anesthesia. Postoperative pain control was achieved using fentanyl and acetaminophen, and the chest drain was removed on postoperative day 1. Fentanyl was discontinued on postoperative day 2, and the patient was discharged on postoperative day 3 and running energetically on postoperative day 4. No complications were observed and no incision problems were noted on postoperative day 23 (Figure 2). A pathological examination revealed ganglioneuroma.

## Discussion

There are several reports of robot-assisted thoracic surgery (da Vinci Xi) in pediatric patients.^1^ The advantages of using a robot include a wide range of articulation, 3-dimensional views, and a comfortable body position. These are considered to be very useful in children with limited working spaces. Disadvantages include a small thorax size that prevents wide placement of the ports causing interference between the forceps, and the robot being too large for operating on children. These procedures were devised and safely performed. The da Vinci SP uses a single port but allows the use of 3 arms (Figure 1, *D*) and a flexible scope. There have been reports of da Vinci SP use in pediatrics; for example, in urology,^2^ but not yet in thoracic surgery. The da Vinci SP overcomes the problem of the distance between ports while maintaining the advantages of the da Vinci Xi. Additionally, they have cosmetic advantages. In thoracic surgery, the maximum port size of 2.5 cm in diameter is too large to be used in the intercostal space, and a subcostal or subxiphoid approach is used,^3^ which may have advantages in children. It is generally considered difficult to remove elastic soft tissue tumors >5 cm in size without intercostal incision, although there have been reports of large tumors being removed through a subcostal incision without intercostal revision.^4^ Funnel chest and thoracic deformities have also been reported as late complications of the intercostal open chest.^5^ This is due to the narrowing of the intercostal space and fusion of the ribs, which causes thoracic deformity to become more prominent as the patient grows, and a left-right difference appears. The da Vinci SP procedure, which does not involve intercostal dissection, has the potential to prevent funnel chest and thoracic deformities occurring in late life. There are disadvantages of using the da Vinci SP for pediatric surgery. As with the da Vinci Xi, these include the large size of the instruments and robot relative to the small size of a pediatric patient, the lack of tactile sensation, and the high cost. In addition, the da Vinci SP does not include devices such as a vessel sealing system, and there are few case reports describing its application in the treatment of children. This requires further development of technology and future follow-up regarding long-term complications. In this case, the tumor was 10 cm in size; however, using the articulated forceps dissection and removal (instead of deployment) of the tumor could be performed, which facilitated a safe surgical procedure. The tumor was also removed by extending the subcostal skin incision up to 5.5 cm. The intercostal area was sandwiched between the hard ribs, but the subcostal incision was soft on the abdominal side, allowing the skin to be stretched and easily removed.

## Conclusions

The da Vinci SP for mediastinal tumors in children has a single incision and does not use the intercostal space, which is a major advantage for patients. Consent to report this case was obtained from the patient and her parents; institutional review board approval was not required.

## Conflict of Interest Statement

The authors reported no conflicts of interest.

The *Journal* policy requires editors and reviewers to disclose conflicts of interest and to decline handling or reviewing manuscripts for which they may have a conflict of interest. The editors and reviewers of this article have no conflicts of interest.

## Supplementary Data

Video 1 Intraoperative video of Da Vinci SP (Intuitive) for mediastinal tumor. Video available at: https://www.jtcvs.org/article/S2666-2507(25)00157-9/fulltext.
