# Supplementary figures and images for: Subcostal approach using the single-port robotic system for a giant ganglioneuroma in a child
Source: JTCVS Tech. 2025 Apr 22;32:141–3. doi: 10.1016/j.xjtc.2025.04.010 (PMC12347245; doi:10.1016/j.xjtc.2025.04.010)

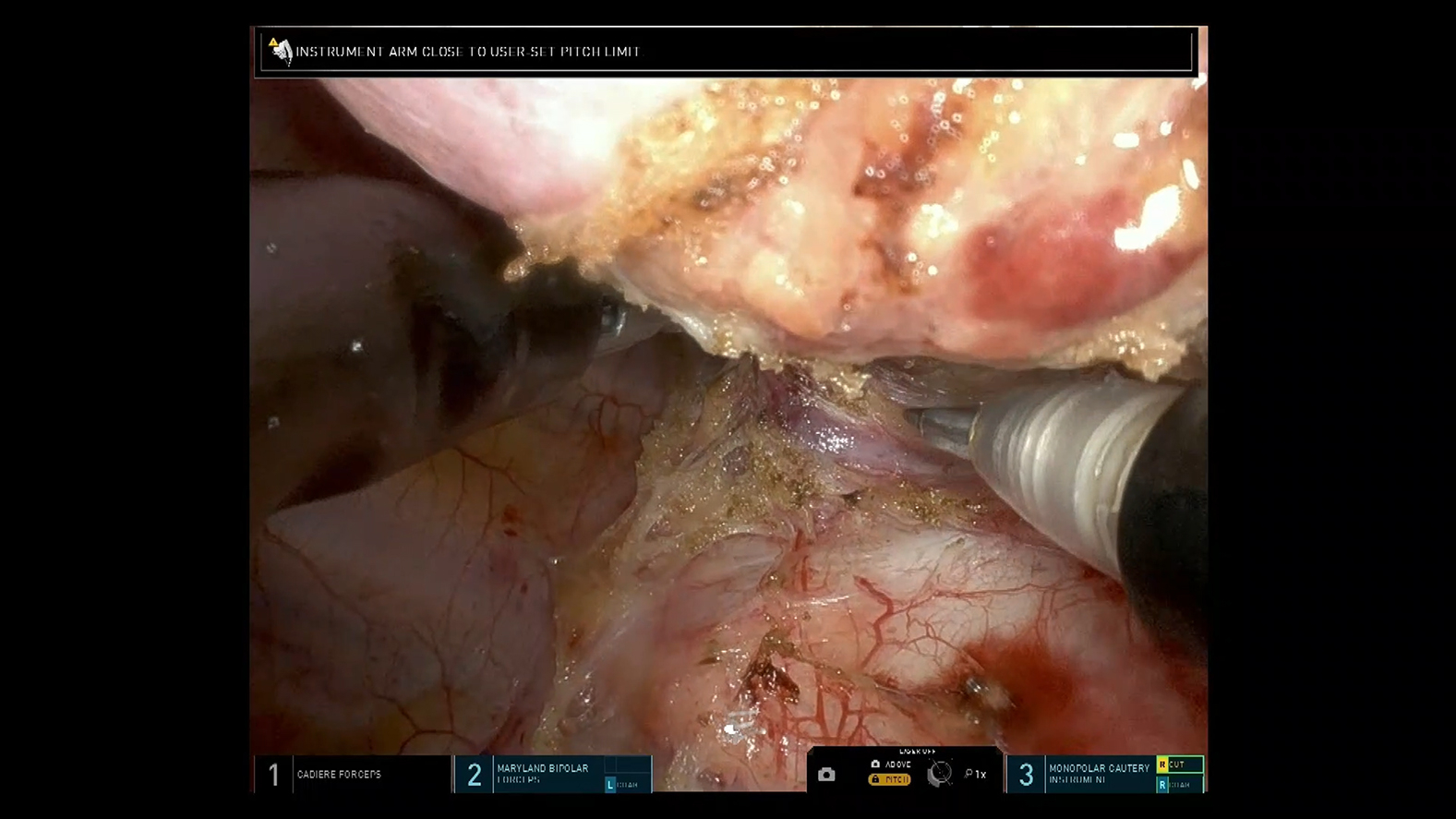

Supplement: Video 1 — Intraoperative video of Da Vinci SP (Intuitive) for mediastinal tumor. Video available at: https://www.jtcvs.org/article/S2666-2507(25)00157-9/fulltext. [file fx2.jpg]
